# Connecting unattached patients to comprehensive primary care: a rapid review

**DOI:** 10.1017/S1463423623000099

**Published:** 2023-03-15

**Authors:** Karina Nabieva, Tess McCutcheon, Clare Liddy

**Affiliations:** 1 Faculty of Medicine, University of Ottawa, Ottawa, ON, Canada; 2 C.T. Lamont Primary Health Care Research Centre, Bruyere Research Institute, Ottawa, ON, Canada; 3 Department of Family Medicine, Faculty of Medicine, University of Ottawa, Ottawa, ON, Canada

**Keywords:** health policy, primary care, unattached patients, literature review

## Abstract

**Introduction:**

Lack of access to primary care providers (PCPs) is a significant hurdle to receiving high-quality comprehensive health care and creates greater reliance on emergency departments and walk-in clinics.

**Methods:**

We conducted a rapid review and analysis of the literature that discusses approaches to increasing access to continuous care for patients with no PCP (‘unattached patients’).

**Results:**

Five distinct themes across 38 resources were identified: financial incentives for patients and providers, health care organization, policy intervention, virtual care and health information technology (HIT), and medical education. Approaches that increased attachment were primary care models that combined two or more of these and reflected the Patient’s Medical Home (PMH) model.

**Conclusions:**

Although there are individual initiatives that could allow for temporary relief, long-term and community-wide success lies in designing models of primary care that use multiple tools, meet the needs of the community, and are supported by regional, provincial, and national policies.

## Introduction

Primary care is associated with numerous benefits for individual and community health, including preventive care, early disease identification and treatment, personalized and relationship-based care, and better management of chronic conditions (The College of Family Physicians of Canada, 2019; McCauley *et al.*, 2021). However, as of 2019, almost 15% of Canadians aged 12 years and over have limited access to a regular primary care provider (PCP) (Government of Canada SC, 2020). This issue affects countries worldwide and has many contributing factors, including population growth, retirement trends, and family medicine graduates choosing not to work in comprehensive family medicine. In Canada, not having a PCP is a significant hurdle to receiving both primary and specialist care, as the gatekeeping nature of the Canadian health care system requires referrals to specialists from a PCP. Consequently, patients with no PCP (“unattached patients”) must seek care through walk-in clinics and emergency departments. This results in poor continuity and coordination of care and unnecessary burdens on the health care system.

Unattached patients are recognized as a growing problem both in Canada and internationally (Cuccia *et al.*, 2019; Nunes and Ferreira, 2019; LExpress.fr, 2020; Kerns and Willis, 2020). Currently in Canada, individuals are responsible for finding a PCP through resources such as centralized waiting lists – a resource managed by provinces where unattached patients who sign up are matched with PCPs through a priority scale and PCP availability (Breton *et al.*, 2015) – or by contacting clinics directly. However, these approaches rely on there being sufficient capacity within the primary care system to absorb new patients.

Several barriers have been identified that affect the number of unattached patients including a lack of family medicine graduates, physicians unwilling to locate to rural regions (Holyk *et al.*, 2017; Jaret, 2020; Campbell, 2021), and a lack of digital tools or multidisciplinary teams that could increase capacity (Holyk *et al.*, 2017; McNeal *et al.*, 2019; Kerns and Willis, 2020). Problems related to PCP shortages and the unequal geographical distribution of PCP practices are well documented internationally (World Health Organization, 2018).

The issue of unattached patients has gained traction beyond researchers and clinicians: the state of physician supply is part of the Canadian Federal Government’s 2021 election platforms (The Liberal Party of Canada, 2021). Additionally, several countries and jurisdictions have launched and embraced new initiatives and approaches to connect individuals to a regular source of comprehensive primary care (Holyk *et al.*, 2017; Cuccia *et al.*, 2019; Nunes and Ferreira, 2019; LExpress.fr, 2020; Kerns and Willis, 2020), yet there is a paucity of research identifying and comparing these initiatives. Further, there is limited universal terminology to describe this patient population, potentially making consolidating and sharing evidence on the topic challenging. Therefore, there is a need to identify existing approaches and their features that lead to successful outcomes in reducing the numbers of unattached patients.

In this article, we review existing literature to uncover and identify themes in approaches to address access to primary care for unattached patients and present two that have been successfully implemented. Through this analysis, we will demonstrate that the most effective interventions are those that address the local practice and policy barriers to facilitate a reformed model of primary care that is capable of providing comprehensive care to a larger patient roster supported by a team. These models are aligned with the framework of the Patient’s Medical Home (PMH) promoted by the College of Family Physicians of Canada. The PMH approach encourages health care teams (e.g., specialists, pharmacists, physicians, social services, etc.) to collaborate to provide individuals with personalized, easily accessible, and coordinated care (The College of Family Physicians of Canada, 2019).

## Methods

In June and July 2021, our team, with the assistance of an academic librarian, conducted a rapid review of the literature to identify approaches to create access to continuous care for unattached patients. Rapid reviews are simplified versions of systematic reviews to produce information in a short period of time, often for the purpose of delivering knowledge synthesis to health care decision-makers, and have no standard methodology (Khangura *et al.*, 2012). We began by searching Medline, PubMed, Google, and Google Scholar using search terms connected by Boolean operators (Box 1). Initial searches yielded primarily Canadian texts, so our team began hand-searching for relevant terms in other countries and contacting Canadian and international researchers in this field. Once we identified new terms, we did a focused Google search of each term. For each search, we scanned the first 10 pages of results. We also identified resources through scanning reference lists and conversations with stakeholders. We included all resources with no limits on publication period that identified an approach to decreasing unattached patient numbers published in English or French. Resources published in any other language were excluded. Through this search, we were able to identify 38 resources that described approaches to decreasing the unattached patient population.

Box 1. Search Strings

- (‘patients without a family doctor’ OR ‘patients without a GP’ OR ‘unattached patient*’) AND (strategy OR solution OR innovati*)
- (‘unattached patient’ OR ‘orphan* patient’) AND (‘family doctor’ OR ‘family physician’ OR ‘primary care’ OR ‘circle of care’ OR ‘family medicine’ OR ‘general pract*’) AND (model OR propos* OR solution* OR polic* OR strateg* OR ‘collaborative care model*’ OR plan OR approach OR clinical informatics OR ‘waiting lists’).
- patient* AND (lacking OR missing OR without) AND (‘medical home’ OR ‘primary care home’ OR ‘patient centered medical home’)
- continuity of care AND (‘family doctor’ OR ‘GP’ OR ‘general practitioner’ OR ‘family physician’ OR ‘primary care physician’) AND (‘new patient*’)

After full-text review of the identified academic and gray literature, we extracted information from each resource on key findings and summarized the intervention. We then conducted an inductive thematic analysis to identify common themes across the resources. Through this process, themes emerged organically from the resources. Two of the authors reviewed the resources independently and met to discuss and compare the themes each had identified. Once they had agreed on preliminary themes, they presented them to our third author for feedback, after which point the five themes presented here were finalized.

## Results

We identified five distinct themes of approaches to reducing the number of unattached patients: financial incentives to patients or providers, health care organization, policy intervention, virtual care and health information technology (HIT), and medical education. Table 1 gives an overview of our findings and conclusions for each.

**Table 1.** Themes of approaches to reduce the number of unattached patients

	Explanation	Example(s)	Conclusion
Financial incentives to patients or physicians	• Implementing policies and practices to encourage attachment by providing financial compensations and/or penalties (Breton *et al.*, 2015; Laberge and Gaudreault, 2015; LExpress.fr, 2020). • Financial incentives for physicians to enroll additional patients, and financial penalties for physicians that do not meet enrollment targets. • Financial penalties to patients for not having a designated physician	• In Quebec, the use of centralized waiting lists has shown that when financial incentives were provided to physicians to enroll vulnerable patients, enrollment for non-vulnerable patients increased to a greater degree than for vulnerable patients (Breton *et al.*, 2015). • Also in Quebec, Bill 20 mandated financial penalties to physicians for not meeting specific enrollment targets and was poorly received by health professionals (Laberge and Gaudreault, 2015). • In France, a financial penalty system for patients was introduced. Patients who did not have a family physician were penalized, with only 30%, instead of 70%, of the cost of consultation being covered (LExpress.fr, 2020).	Although financial incentives may work on a smaller scale, our search found that they do not appear to significantly increase patient attachment.
Better health care organization	• Reorganizing clinics can increase capacity for a more extensive patient roster. • Implementing interprofessional health care teams to deliver team-based care can increase care capacity. Delegating certain physician duties to other health care professionals (e.g., physician assistants, nurses, nurse practitioners, pharmacists, social workers, and paramedics) and delivering care in alternative settings, not only in one main clinic (Foy and Hahn, 2009; Virani, 2012; Cuccia *et al.*, 2019; McNeal *et al.*, 2019). • Using virtual care methods often supports collaborative methods of health system organization (Bodenheimer and Smith, 2013; Green *et al.*, 2013; Ware and Mawby, 2015; Holyk *et al.*, 2017; McNeal *et al.*, 2019; Kerns and Willis, 2020).	• In Costa Rica, during yearly visits, patients have access to a team of medical professionals (a physician, a nurse, a technical assistant, a medical data clerk, and a pharmacist) (Cuccia *et al.*, 2019). All care from preventative care to pharmacy needs is coordinated between team members (Cuccia *et al.*, 2019). • In Australia, one initiative proposed implementing a virtual community of practice linking urban and rural physicians to foster a sense of community and to share skills (Ortolan, 2021). This was in response to the common barrier of finding physicians willing to locate to rural communities.	Reorganizing health care teams and processes to rely on multidisciplinary teams has proven to be effective, especially when virtual technologies are used to promote team-based integrated care (Holyk *et al.*, 2017; McNeal *et al.*, 2019).
Policy intervention	• Policy changes have been implemented to address the challenges linked to unattached patients. Examples of such policies are as follows: growing the supply of physicians by increasing the number of primary care residency spots, student loan forgiveness, changing scope of practice for nonphysicians, and payment model reform to support multidisciplinary team-based care (Carrier *et al.*, 2011).	• In British Columbia, the ‘Attachment Initiative’ is a provincial policy initiative to support several local approaches to increase patient attachment to primary care by increasing clinic capacities and targeting vulnerable patients (Webb, 2016; Vancouver Division of Family Practice, nd). • In Canada and the United States, expanding nonphysician health care professionals’ scope of practice could increase the supply of health care services and likely help meet the demand for PCPs significantly (Carrier *et al.*, 2011; Poghosyan *et al.*, 2017; Globerman *et al.*, 2018).	The policy interventions that show promise are centered on supporting communities to implement initiatives that are tailored to the local context (Webb, 2016; Holyk *et al.*, 2017; Cuccia *et al.*, 2019; Vancouver Division of Family Practice, nd). Through this search, we found resources describing policy interventions, such as payment model and scope of practice reform to promote multidisciplinary clinics, but there were limited resources describing the implementation of such policies.
Virtual care and HIT	• Utilizing technology to enhance patient access, increase care teams’ capacity, and facilitate better data collection for research and quality improvement initiatives facilitates patient attachment.	• In Almeda County (San Francisco, United States), an emergency department proposed the use of EPIC, an electronic medical record platform, to generate a daily report identifying patients lacking a regular PCP and assisting them in finding care (Rodriguez, 2015). • Other examples identified include monitoring patient panels through utilization data and auto-assigning patients to less active panels (Marx *et al.*, 2009; Balasubramanian *et al.*, 2010), online referral systems for patients visiting an emergency room (Murnik *et al.*, 2006), a website where patients can locate available practitioners (NHS, nd), and virtual care delivery at satellite clinics using video or phone visits (Holyk *et al.*, 2017; McNeal *et al.*, 2019).	Though such interventions can be helpful to facilitate a connection, there needs to be an adequate supply of PCPs to be able to meet the demand (Breton *et al.*, 2018).
Medical education	• Rethinking medical school education can increase the supply of physicians to meet the demand.	• Universities such as Oregon Health & Sciences University School of Medicine, University of Kansas School of Medicine, the University of California School of Medicine, and Dalhousie University in Canada recruit candidates from rural communities and facilitate rural placements to maximize the chances of students working in these communities upon graduation (Jaret, 2020; Campbell, 2021). • In Portugal, another approach implemented was to hire more recent graduates, though this approach did not have long-term success (Nunes and Ferreira, 2019). • Other strategies include changing the culture of primary care by promoting multidisciplinary team-based approaches in health professional education (McNeal *et al.*, 2019).	Rural medical education initiatives have shown preliminary success at increasing primary care providers in rural settings (Jaret, 2020; Campbell, 2021). More initiatives are required to teach medical professionals to successfully work in multidisciplinary teams (McNeal *et al.*, 2019).

By analyzing various health care interventions worldwide, we found that the most promising interventions were primary care models that combined at least two of the identified themes and reflected the PMH model. One intervention that stood out was by Carrier Sekani Family Services (CSFS), located in rural British Columbia, Canada (Holyk *et al.*, 2017), where they have implemented compelling solutions to multiple difficulties encountered by patients accessing primary care. For instance, the geographical area is vast with many people living in hard-to-access, remote areas that previously relied on fly-in physicians. To address this, the CSFS introduced satellite clinics run by nurse practitioners, where family physicians provide regular care to patients through telemedicine and occasional in-person appointments. Additionally, individuals may visit different clinics in the area and have their health information shared seamlessly among providers through a centralized electronic medical record. The use of a multidisciplinary team in a hub and spoke model along with effective health information technologies allows the clinics to maximize workforce capacity and extend patient rosters. This is an example of how primary care appears to be most successful when developed based on specific community needs.

The CSFS model demonstrates the ability to increase primary care capacity while providing care that reflects the concept of the PMH model. Though not all patients using CSFS identify the clinic as their primary care medical home (Holyk *et al.*, 2017), the number of clinic visits rose from 3471 in 2018 to 4394 in 2019 (Carrier Sekani Family Services, 2019). Additionally, a study on a sample of patients using the clinic found that continuity of care increased, while reliance on emergency departments decreased (Holyk *et al.*, 2017).

Other successful interventions we found were similar in that the pillars of the PMH model were addressed while ensuring the intervention was grounded in the specific culture of the community. For example, the Patient Aligned Care Team (PACT) implemented by the Veterans Health Administration in the United States is focused on providing flexible team-based care that meets the unique social and health needs of veterans (Schuttner *et al*., 2020). In the PACT model, health information technologies are also used to facilitate more virtual encounters and increase communication between providers, patients, and families. In the first two years of implementation, the number of enrolled patients increased from 4 817 272 to 5 163 531 (Rosland *et al.*, 2013).

## Discussion

This rapid review provides an identification and analysis of approaches to reducing the numbers of unattached patients taken worldwide and describes two unique programs that show promising results in tackling this issue. We found that these approaches fall into five distinct themes: financial incentives to patients or providers, health care organization, policy intervention, virtual care and HIT, and medical education.

In Canada, there is a growing number of unattached patients, yet this population has been largely left out of the conversation when it comes to providing efficient, effective health care. The current review discusses why unattached patients have proven to be such a challenge, and how Canadian health care practitioners and policymakers can work toward addressing these challenges while simultaneously increasing access to quality primary care services across the country.

While Canada has experimented with various solutions to alleviate issues associated with the PCP shortage and unattached patients, reforms are needed to address the organization of a primary care system within a community context (Schuttner *et al*., 2020). For a health care delivery model to be successful in delivering equitable access to all community members it serves, meaningful, multilevel interventions that are aligned with and mutually supportive of each other are needed. This includes those from governments that introduce policies to reform payment structures and increase the scope of practice for nonphysician health care providers (Kirchner *et al.*, 2010; McCauley *et al.*, 2021). In addition, changes are needed at the clinic level to address the way providers work together, such as the use of HIT for better integration of care, reorganization of the health care system favoring multidisciplinary team-based care, and care delivery tailored to the local population (Holyk *et al.*, 2017; McCauley *et al.*, 2021).

These findings reflect changes needed to the primary care system outlined in a recent report by the National Academies of Sciences, Engineering, and Medicine (McCauley *et al.*, 2021). The authors highlight the need for interventions at local, state, and national levels to incorporate care that is person-centered, integrated, and sustained through relationships between care providers, patients, families, and communities (McCauley *et al.*, 2021). To achieve changes at a local level, policies are needed that allow for regional approaches. For example, Ontario has implemented Ontario Health Teams, encouraging health care approaches that apply to the population’s needs and preferences in segmented geographic regions (Ontario Health Teams, nd).

The findings from this rapid evidence review show a heterogeneous body of literature. More research is needed to highlight the innovative models of primary care that increase attachment by taking a holistic, community-based approach. Furthermore, there is also a need for critical evaluations of the models in existence to establish evidence-based approaches to decreasing rates of unattached patients at all levels of implementation. We found that the terminology used to describe the topic is vast and varying. For this reason, we recommend that future attention be put toward synthesizing and understanding the terminology used to be better able to identify and learn from innovative models that have proven successful.

While we have focused our findings on the Canadian context, many other countries are facing similar challenges regarding unattached patients (Corscadden *et al.*, 2018), so this type of review is of international import. The benefit of looking to other countries for possible answers to health care delivery and policy questions is that it broadens the scope of possibilities beyond a country’s historical, cultural, and structural contexts, wherein certain blind spots are intrinsic and therefore unavoidable. However, due to these same contexts, some policy approaches will not translate or see the same successes from one country to another (Groenewegen *et al.*, 2013). For example, the success of the intervention we described by CSFS relied on the use of multidisciplinary health teams (Holyk *et al.*, 2017). While this approach worked in the Canadian context where PCPs serve a gatekeeping function to accessing health care services, patients living in countries where PCPs do not serve this centralized role report less satisfaction and worse care when multiple physicians and health services are co-located (Bonciani *et al.*, 2018).

## Conclusion

Although there are individual initiatives, like centralized waiting lists, that could allow for temporary relief, our findings suggest that long-term and community-wide success lies in the design of models of primary care that use multiple tools, not just the adoption of one single element. Further, models such as the CSFS have proven to be successful in attaching patients to PCPs, and interventions must be holistically grounded in the target community. Focusing on reforming models of primary care delivery to meet community needs, rather than changing one specific element, could result in care that is based on a community’s unique needs and preferences, dependable throughout the lifespan, and, most importantly, integrated across all health care services. Future research should be focused on identifying, learning, and adapting elements from successful models of primary care that are grounded in the community.
